# *Ex Vivo* Tracer Efficacy in Optical Imaging of *Staphylococcus Aureus* Nuclease Activity

**DOI:** 10.1038/s41598-018-19289-y

**Published:** 2018-01-22

**Authors:** Colin W. K. Rosman, Francisco Romero Pastrana, Girbe Buist, Marjolein Heuker, Marleen van Oosten, James O. McNamara, Gooitzen M. van Dam, Jan Maarten van Dijl

**Affiliations:** 1 Department of Medical Microbiology, University of Groningen, University Medical Center Groningen, Groningen, The Netherlands; 2 Department of Surgery, University of Groningen, University Medical Center Groningen, Groningen, The Netherlands; 3 Department of Biomedical Engineering, University of Groningen, University Medical Center Groningen, Groningen, The Netherlands; 4 Department of Internal Medicine, Roy J. and Lucille A. Carver College of Medicine, University of Iowa, Iowa City, Iowa USA

## Abstract

The key to effective treatment of bacterial infections is a swift and reliable diagnosis. Current clinical standards of bacterial diagnosis are slow and laborious. There are several anatomical imaging modalities that can detect inflammation, but none can distinguish between bacterial and sterile inflammation. Novel tracers such as smart activatable fluorescent probes represent a promising development that allow fast and specific testing without the use of ionizing radiation. Previously, a smart activatable probe was developed that is a substrate for the micrococcal nuclease as produced by *Staphylococcus aureus*. In the present study, the function of this probe was validated. Practical applicability in terms of sensitivity was assessed by incubation of the probe with 26 clinical *S*. *aureus* isolates, and probe specificity was verified by incubation with 30 clinical isolates and laboratory strains of various bacterial pathogens. The results show that the nuclease-specific probe was activated by all tested *S*. *aureus* isolates and laboratory strains with a threshold of ~10^6^–10^7^ cells/mL. The probe was also activated by certain opportunistic staphylococci. We therefore propose that the studied nuclease probe represents a significant step forward to address the need for a rapid, practical, and precise method to detect infections caused by *S*. *aureus*.

## Introduction

Bacterial infections are a serious clinical problem in all fields of modern medicine. *Staphylococcus aureus* is one of the largest contributors to in-hospital infections, causing 19.5% of all surgical site infections (SSI’s), 14.6% of all Intensive Care Unit (ICU)-acquired pneumonia episodes and 10.1% of all ICU-acquired bloodstream infections in the European Union^1^. What makes this particular pathogen so dangerous is the rapid acquisition of resistance and the ability to form biofilms. *S*. *aureus* infections, and especially methicillin-resistant *S*. *aureus* (MRSA), are hard to treat because of these properties. In 2014, 46.4% of all *S*. *aureus* associated with ICU-acquired infections were MRSA^1^.

The most common sites of infection for *S*. *aureus* are catheters, prosthetic devices and SSI’s^2^. SSI and infection of prosthetic devices are especially difficult to treat once biofilm formation has started. *S*. *aureus* is also a major cause of many focal bacterial infections, such as osteomyelitis^3^, septic arthritis^4^, and pyomyositis^5^. What makes the treatment of these infections even more challenging is that these patients are often old, morbid and stationary.

The current diagnostic standards are based on sample collection by, respectively, performing a biopsy or swabbing the affected area and culturing these samples. Downsides of both approaches are the amount of time and labor it takes to reach a reliable diagnosis, and the possible harm to the patient^6,7^. Moreover, the different diagnostic techniques are prone to false-negative results^8^. Importantly, focal bacterial infections can be life-threatening, while current diagnostic procedures typically take hours to days, which gives rise to higher hospital costs and patient dissatisfaction.

Other ways of detecting infections are the use of anatomical imaging modalities, such as ultrasound, computed tomography (CT) or magnetic resonance imaging (MRI), or functional imaging modalities, such as positron emission tomography (PET), single photon emission computed tomography (SPECT) or scintigraphy^9,10^. These techniques allow detection in the whole body and are noninvasive. However, none of these imaging modalities can discriminate a bacterial inflammation from a sterile inflammation, nor can they give any information about the type of pathogen^11–14^.

In recent years, multiple researchers have proposed techniques to improve these imaging modalities by using a wide array of probes and suggesting new imaging modalities^15^. Promising new imaging techniques are fluorescent imaging and photoacoustic tomography (PAT)^16^. These techniques do not require any form of ionizing radiation like CT, MRI, PET, SPECT and scintigraphy. They can also be used relatively easily in the operating theatre. The main advantage of PAT is that the optical scattering is bypassed by using soundwaves^15^. However, before it can be used in the clinic both the PAT capabilities and probe functionality have to be improved^17^.

To be able to distinguish between sterile inflammation and bacterial infections various probes have been designed that specifically target bacteria. Examples are tracers linked to antibiotics, such as vancomycin^18^, or sugars that are solely metabolized by bacteria, such as sorbitol^19^ and maltose^20,21^. These types of tracers do not allow identification of a specific pathogen, but give an indication of the type of bacteria causing infection. An even more specific approach involves the use of fluorescently labeled antibodies or nanobodies. In general, it takes approximately 4–6 days for antibodies to reach the optimal target to normal tissue (T/N) ratio, which makes the use of antibodies less practical when rapid diagnosis is needed^22^. Researchers have developed nanobodies that reach the optimal T/N ratio in several hours to overcome this problem^23^. First results look promising, though much more extensive research is needed to safeguard the function after binding to imaging agents and reduce clearance^23^.

A review on biomarkers in wound infections has been published recently^24^. The authors of this article concluded that there is an urgent need for novel diagnostic tools in wound infection. There are multiple promising discoveries in this field of research, though it seems that the rapid diagnostic techniques still lack specificity and the specific diagnostic techniques are laborious and time-consuming.

Smart activatable probes represent a highly innovative development. After activation, these probes emit a different signal from their default state. By determining the T/N ratio the accuracy of the measurements can be vastly improved^25,26^. Degradative enzymes make optimal targets for this type of probe. A promising enzyme is the micrococcal nuclease, which is a DNA-degrading enzyme of which many different types of bacteria express their own, type-specific version^27^. In particular, Hernandez *et al*. have reported a nuclease-activated probe that reacted specifically to Staphylococcal nuclease^28^. This so-called ‘TT-probe’ consisted of a fluorophore and a quencher that are connected by a short, 11-base, oligonucleotide. Specifically, the TT-probe used fluorescein amidite (FAM) as a fluorophore, 2 deoxythymidine nucleotides flanked by several 2′-O-methyl modified nucleotides as oligonucleotide and the ZEN and Iowa Black RQ quenchers of Integrated DNA Technologies (IDT). For *in vivo* experiments the FAM was replaced by Cy5.5. The latter probe allowed the detection of a focal *S*. *aureus* infection within 1 hour *in vitro* and 1–2 hours in mice.

The present study was aimed at validating the function and determining the potential for clinical application of the nuclease-activated P2&3 TT-probe. The P2&3 TT-probe is a derivative of the original TT-probe^28^ in which the unmodified TT dinucleotide is placed at nucleotide positions 2 and 3 of the 11-nucleotide-long oligonucleotide (versus positions 5 and 6 in the original TT-probe). This probe derivative was found to yield greater sensitivity for micrococcal nuclease of *S*. *aureus* than the original TT-probe (McNamara lab, unpublished data). We focused on verification of the working mechanism, probe stability in human blood, sensitivity and specificity in a clinical setting. The specificity and sensitivity were determined using clinical isolates of *S*. *aureus* and related bacterial species.

## Results

### Probe activation by secreted nuclease

**Figure 1:**
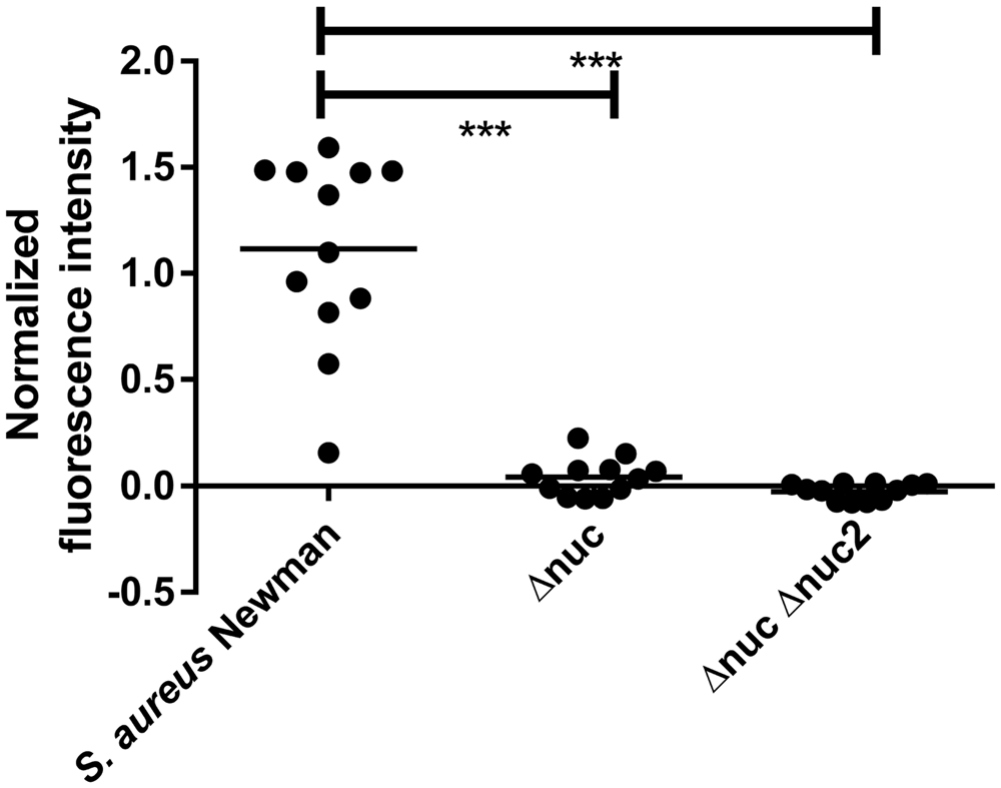
P2&3 TT-probe activation by *S*. *aureus* Newman (Newman), *S*. *aureus* Newman Δ*nuc* (Δnuc) and *S*. *aureus* Newman Δ*nuc*, Δ*nuc2* (Δnuc, Δnuc2). The horizontal line represents the mean. ***Indicates P < 0.0001.

The P2&3 TT-probe was incubated with 3 different *S*. *aureus* Newman strains, namely the wild-type, a Δ*nuc* mutant and a Δ*nuc* Δ*nuc2* double mutant. Correction of background was based on the negative control and normalization was based on the positive control. As shown in Fig. 1, probe activation was almost solely dependent on secreted nuclease. Incubation of the Newman wild-type strain with the P2&3 TT-probe resulted in a mean fluorescence intensity of 1.115(sD 0.448), incubation of the Δ*nuc* mutant in a mean fluorescence intensity of 0.0421(sD 0.088), and the Δ*nuc* Δ*nuc2* double mutant in a mean fluorescence intensity of −0.251 (sD 0.038). After incubation both nuclease mutants showed statistically different probe activation levels compared to the wildtype (P < 0,0001, P < 0,0001). Probe activation was not statistically different in both mutants.

### Probe stability in human blood and plasma

**Figure 2:**
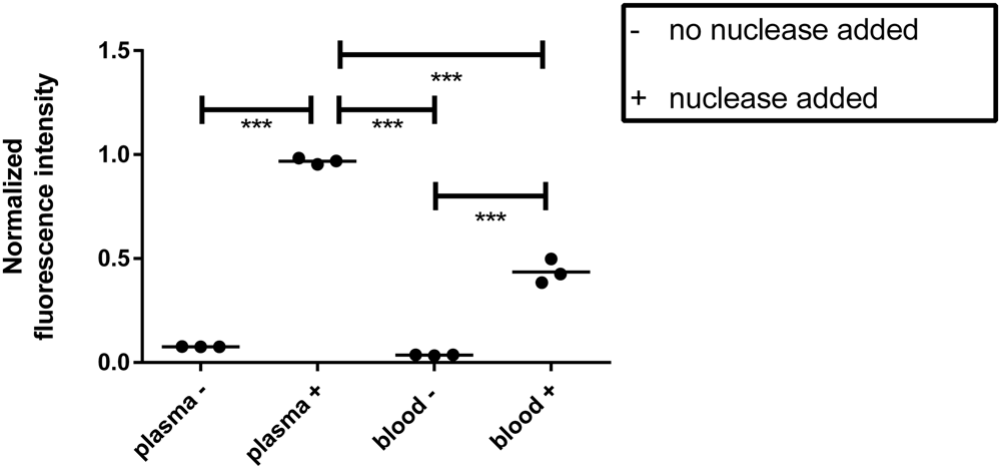
Normalized fluorescence intensity after probe incubation in human blood and plasma. Horizontal line represents the mean. ^–^indicated no nuclease was added. ^+^indicates nuclease was added. **Indicates P < 0.001, ***indicates P < 0.0001.

Incubation of the P2&3 TT-probe in human plasma or human blood did not result in probe activation. In contrast, incubation of the probe in human plasma or blood with purified nuclease resulted in activation of the probe (Fig. 2). Correction of background was based on the negative control and normalization was based on the positive control. Incubation of plasma without nuclease resulted in a fluorescence intensity of 0.076 (sD 0.000), plasma with nuclease in a fluorescence intensity of 0.969 (sD 0.015), blood without nuclease in a fluorescence intensity of 0.035 (sD 0.002), and blood with nuclease in a fluorescence intensity of 0.436 (sD 0.058). Incubations with nuclease were statistically different from incubations without nuclease (plasma P < 0.0001; blood P < 0.001). Importantly, a significant difference in P2&3 TT-probe activation between incubations of plasma and blood with nuclease was observed (P < 0.0001). No statistical difference between incubations of plasma or blood without nuclease was found. The lower fluorescence intensity of activated probe in blood was expected due to overlap in the absorption spectrum of hemoglobin and the emission spectrum of FITC.

### Sensitivity of the nuclease probe

**Figure 3:**
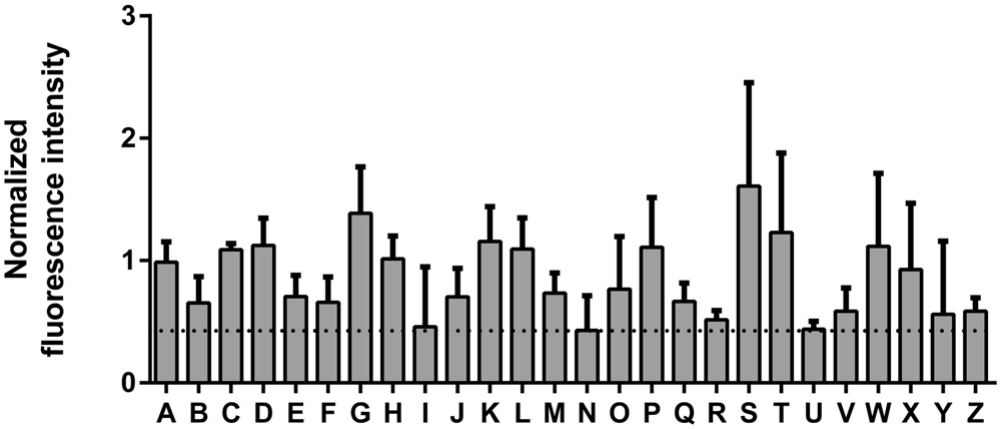
Normalized fluorescence intensity after incubation of clinical *S. aureus* isolates. A/Z indicate the isolates that were incubated. The dotted horizontal line represents the positive cutoff. Bars indicate mean fluorescence. Capped lines indicate standard deviation.

26 clinical *S*. *aureus* isolates were incubated with the P2&3 TT-probe in triplicate. Correction of background was again based on the negative control and normalization on the positive control. The mean normalized fluorescence intensity was 0.86 (sD 0.43). The cutoff for a positive reaction was set at 43% of the positive control signal. This cutoff point represents the mean activation minus one standard deviation and covers all tested *S*. *aureus* strains (Fig. 3). Of note, a large heterogeneity in probe activation was detected. This corresponds with the heterogeneity observed in nuclease production as documented in a previous study where the exoproteomes of 25 of the presently tested clinical *S*. *aureus* isolates were characterized^29^.

**Figure 4:**
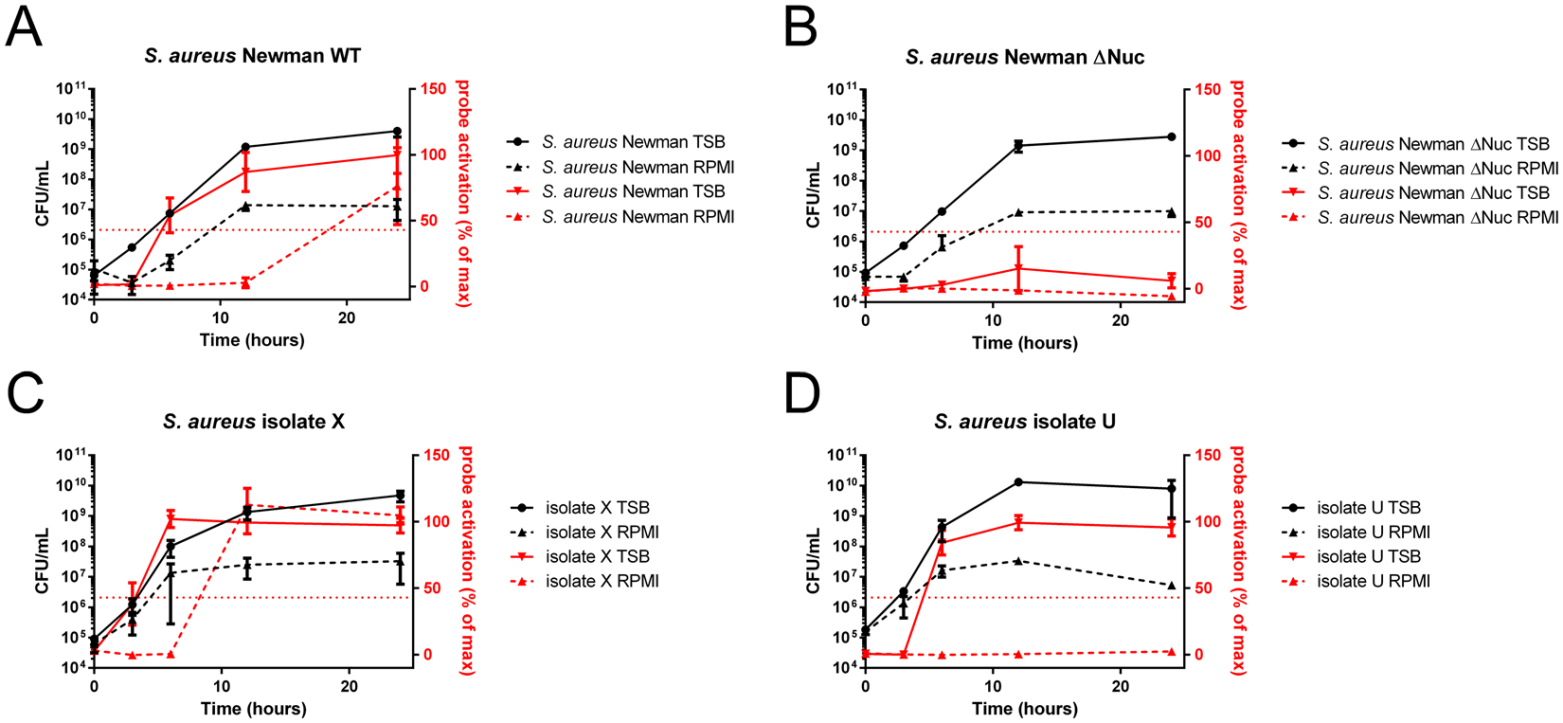
Activation of the P2&3 TT-probe by *S*. *aureus* grown to different stages in TSB and RPMI. Left Y-axis and lines in black indicate CFU per mL, right Y-axis and lines in red indicate probe activation as a percentage of the maximum activation. All experiments were done in triplicate, and the plots present the average numbers of the measurements. Capped lines indicate standard deviation. Strains included were: A, *S*. *aureus* Newman wild-type; B, nuclease deficient *S*. *aureus* mutant (Δ*nuc*); C, *S*. *aureus* clinical isolate X^29^; and D, *S*. *aureus* clinical isolate U^29^.

For validation of P2&3 TT-probe activation by different amounts of bacteria or by bacteria grown to different growth stages, we measured the nuclease activity of four *S*. *aureus* strains. These included *S*. *aureus* Newman wild-type, the nuclease-deficient Δ*nuc* mutant, and the clinical isolates X and U. The two clinical isolates were chosen, because of their differing nuclease production levels and different clinical backgrounds. Isolate X is a high-level nuclease producer that was collected from the throat of a patient with cholangitis^9^. Isolate U is a low-level nuclease producer that was collected from the blood of a patient with sepsis, meningitis, and probably endocarditis^9^. All bacteria were grown in tryptic soy broth (TSB) and Roswell Park Memorial Institute medium (RPMI). Of note, the RPMI 1640 was used to grow *S*. *aureus*, because the global gene expression patterns of *S*. *aureus* cells grown in RPMI or human plasma were recently shown to be highly similar^30^. At T = 0, 3, 6, 12, and 24 hours, the colony-forming units (CFU) per mL and nuclease activity were measured (Fig. 4). All strains grew more abundantly in TSB than in RPMI medium. As shown in Fig. 4, the wild-type Newman strain showed an increase in nuclease activity upon entering the stationary growth phase, while no nuclease activity was detectable for the isogenic Δ*nuc* mutant. As expected, isolate X showed significant nuclease production in the late exponential and stationary growth phases (Fig. 4C). In contrast, isolate U showed significant nuclease activity during growth in TSB, but it did not show any nuclease activity whilst growing in RPMI medium. With exception of this specific condition, and the nuclease deficient mutant, all strains activated the probe above the threshold at 10^6^−10^7^ CFU/mL, regardless of the growth medium used. Essentially the same results were obtained when the nuclease activity in the samples was measured with the so-called poly T probe (Supplemental Fig. 1). This poly T probe consists of the same fluorophore and quencher as the P2&3 TT probe, but the connecting oligonucleotide consists of 11 unmodified thymine bases^31^. This results in a higher sensitivity for nuclease activity which enables detection of the lower level of nuclease in isolate U when it is grown in RPMI, but lowers the specificity to Staphylococcal nuclease as the poly T probe can be degraded by many different endonucleases^28^.

### Specificity of the nuclease probe

**Figure 5:**
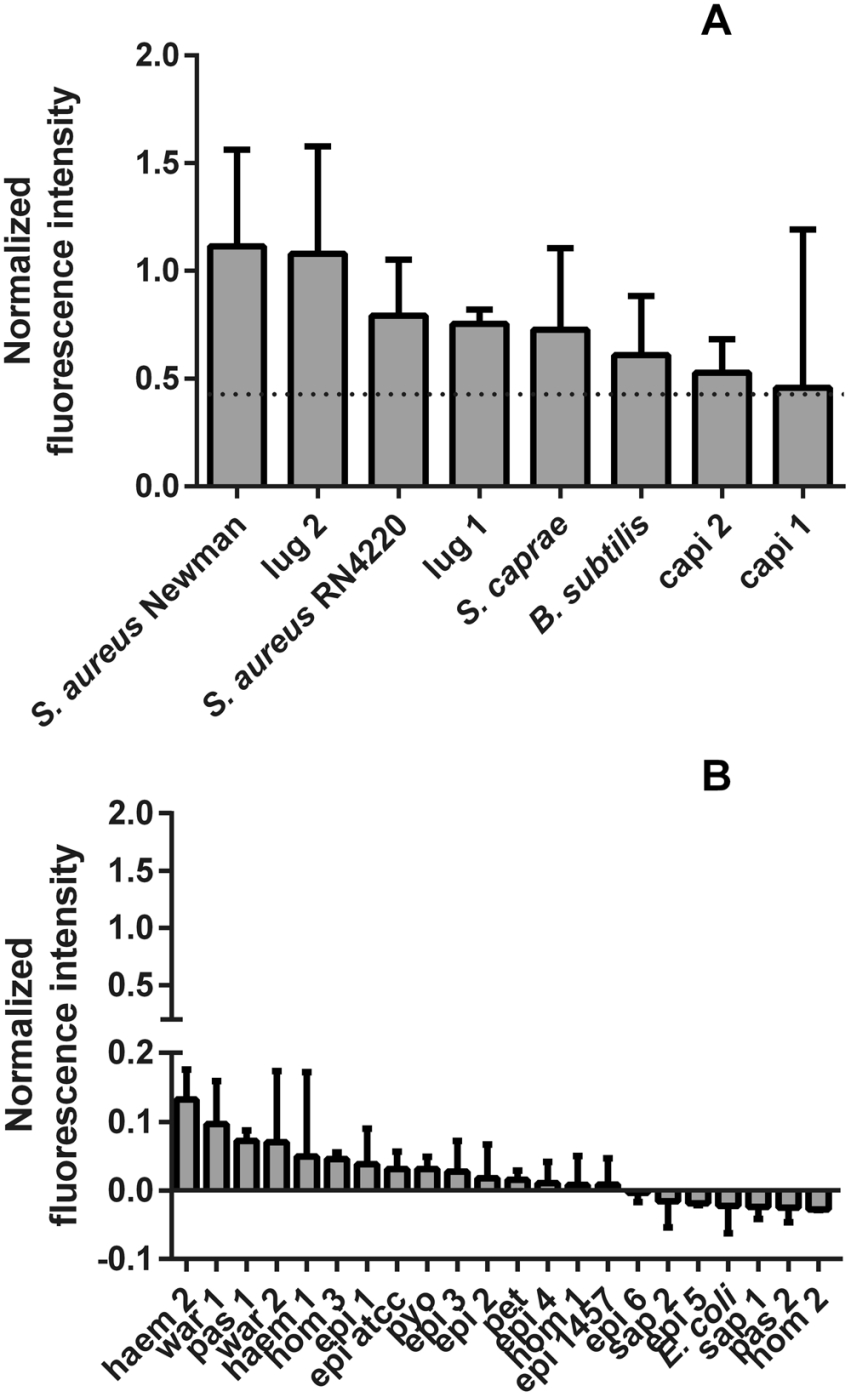
P2&3 TT-probe activiation by different bacterial isolates. (**A**) Normalized fluorescense intensity upon P2&3 TT-probe activation by of all strains that tested positive. *B*. *subtilis* was grown in LB and the respective measurements were normalized with LB controls. All other strains were grown in TSB. (**B**) normalized fluorescence intensity of strains that tested negative with the P2&3 TT-probe. *E*. *coli* was grown in LB and the respective measurements were normalized with LB controls. All other strains were grown in TSB.

To verify specificity of the P2&3 TT-probe, thirty different bacterial isolates were incubated with this probe. These included two *S*. *aureus* laboratory strains (Newman and RN4220), four non-staphylococcal laboratory strains (*Bacillus subtilis* 168, *E*. *coli* DH5α, *Staphylococcus epidermidis* ATCC 35984 and *S*. *epidermidis* 1457), and a total of 24 clinical isolates of 12 different species (see Materials and Methods). Abiding by the cutoff point that was previously determined, eight bacterial isolates tested positive for nuclease activity. These were *S*. *aureus* Newman, *S*. *aureus* RN4220, *Staphylococcus lugdunensis* 1 and 2, *Staphylococcus caprae*, *Staphylococcus capitis* 1 and 2, and *B*. *subtilis* 168 (Fig. 5A). The 22 other strains tested negative with values lower than the cutoff point (Fig. 5B).

## Discussion

The objective of this research was to validate the function of the nuclease-activated P2&3 TT-probe, an optimized version of the probe described by Hernandez *et al*.^28^, and to assess its *ex vivo* efficacy in optical imaging of *S*. *aureus* infections. All incubations were done in 1 hour, demonstrating the swiftness of the process. As expected, the P2&3 TT-probe was activated by secreted nuclease of *S*. *aureus*. The probe proved to be stable enough to maintain its function in both human plasma and blood. The P2&3 TT-probe was also shown to be activated upon incubation with a wide variety of clinical *S*. *aureus* isolates for the first time. Apart from *S*. *aureus*, incubation with four other staphylococcal species also resulted in activation of the probe.

The P2&3 TT-probe was shown to be stable in both human blood and human plasma. However, the signal strength was much lower in human blood than in plasma. This is probably caused by the overlap of the FITC label emission spectrum (521 nm) and the absorption spectrum of hemoglobin^32^. Nevertheless, *in vitro* absorption of the P2&3 TT-probe’s fluorescence does not have to pose a problem, as long as the normalization standard is subject to the same degree of absorption. For *in vivo* use, or use in an assay that includes hemoglobin a different fluorophore will have to be used. For example, imaging *in vivo* with a Cy5 fluorophore instead of FITC was already done by Hernandez *et al*.^28^.

The clinical *S*. *aureus* isolates tested are representative for the diversity of *S*. *aureus* that can be encountered in a clinical setting, in our case the University Medical Center Groningen. Our results thus show that the probe is an effective tool to detect the presence of such different *S*. *aureus* isolates. The signal strength observed in the different *S*. *aureus* incubations showed a great heterogeneity, as was to be expected based on previous proteome analyses that had shown variety in the levels of nuclease produced by different clinical *S*. *aureus* isolates^29^. This heterogeneity implies that nuclease production, and therefore probe activation, is likely to be different for every *S*. *aureus* strain.

Three of the four non-*S*. *aureus* species that tested positive are regarded as harmless, being soil bacteria (*B*. *subtilis*) and non-pathogenic human commensals (*S*. *capitis* and *S*. *caprae*). The fourth bacterium that tested positive is *S*. *lugdunensis*. This species is regarded as a true pathogen and has been implicated in infections reminiscent of *S*. *aureus* infections^33^. Activation of the TT-probe by *S*. *lugdunensis* was also reported by Hernandez *et al*.^28^. We note that Hernandez *et al*. and Brakstad *et al*. 1995 found relatively lower levels of nuclease activity produced by *S*. *lugdunensis* (Hernandez *et al*.), *S*. *capitis* (Brakstad *et al*.) and *S*. *caprae* (Brakstad *et al*.) as compared to *S*. *aureus*^34^. As most of the signals in the positives of Fig. 5A are essentially saturated, it is difficult to derive relative levels of nuclease activity from these samples. Our results are thus not inconsistent with these previous reports. Of note, all tested bacterial species that can cause infection gave a positive signal with the probe, while bacterial species that produce non-micrococcal nucleases did not activate the probe. This indicates that the tested P2&3 TT-probe has a high application potential in a clinical-diagnostic setting. One concern could be that mutations in the nuclease-encoding gene could cause a diagnostic resistance. To date, there has been no large investigation on the possible occurrence of nuclease-deficient *S*. *aureus* in the clinic. However, nuclease plays an important role in *S*. *aureus* biofilm dynamics and has been widely used as a specific marker for *S*. *aureus*. Thus, it seems unlikely that spontaneous nuclease mutations will occur. Two other conceivable complications are (i) that it is not precisely known how nuclease production is influenced by random mutations that can be encountered in a clinical setting, or (ii) what influence different growth conditions may have on nuclease production. The lack of P2&3 TT-probe activation by the clinical *S*. *aureus* isolate U grown in RMPI medium is an example of the latter possibility. Here, it should be noted that isolate U is the clinical isolate with the lowest nuclease production levels in our collection.

Altogether, our present observations support the view that smart-activatable probes are a promising development towards rapid, robust, sensitive and specific diagnostic and imaging tools. Importantly, the presently studied P2&3 TT-probe allows for specific *in vivo* imaging of nucleases produced by pathogenic staphylococci without the use of ionizing radiation. Future use of this probe could be in clinical diagnostic tools or in the context of an imaging modality. Clinical applications may include the identification of *S*. *aureus* on patient material (like skin or bodily fluids), material that has been in contact with patients (like bandages, prosthetics or catheters) or even *in vivo* imaging during and after surgery. Additional research will be needed to investigate all possible future applications.

## Materials and Methods

### Bacterial lab strains and clinical isolates

The *S*. *aureus* Newman nuclease mutants were a gift from Dr. Alexander Horswill, Department of Microbiology, Roy J. and Lucille A. Carver College of Medicine, University of Iowa (USA). The clinical strains were collected at the Department Medical Microbiology (bacterial diagnostics) in the University Medical Center Groningen (UMCG) and coded to ensure patient privacy. Clinical *S*. *aureus* isolates (n = 26) are coded with a letter. Isolates A/Y were collected in a previous study^29^. Isolate Z was collected later. The other clinical strains are shown as species and number. This number is used to differentiate between independent isolates of the same species. These include *S*. *epidermidis* (epi 1–6), *Staphylococcus hominis* (hom 1–3), *Staphylococcus haemolyticus* (haem 1–2), *Staphylococcus pasteuri* (pas 1–2), *Staphylococcus warneri* (war 1–2), *Staphylococcus saprophyticus* (sap 1–2), *S*. *lugdunensis* (lug 1–2), *S*. *capitis* (capi 1–2), *S*. *caprae* (caprae), *Staphylococcus pettenkoferi* (pet) and *Streptococcus pyogenes* (pyo). The laboratory strains used were: *B*. *subtilis* 168, *S*. *aureus* Newman^35^, *S*. *aureus* RN4220, *Escherichia coli* DH5α, *S*. *epidermidis* American type culture collection (ATCC) 35984 and *S*. *epidermidis* 1457. Before use all bacterial strains were stored at −80 °C in a 10% glycerol suspension.

### Growth media

All bacteria were cultured by plating on a blood agar with 5% sheep blood (BA; Media Products B.V., Groningen, the Netherlands). After plating the bacteria were incubated overnight in a stove at 37 °C. Then one colony was resuspended in a round bottom 96-well plate with 200 µL growth medium. *B*. *subtilis* and *E*. *coli* were grown in Lysogeny Broth (LB; BD Biosciences, Sparks, MD). All other bacteria were grown in TSB (BD Biosciences, Sparks, MD). After inoculation, the 96-wells plate was placed in an orbital shaker-incubator and incubated for 1 hour at 37 °C at 800 rpm. RPMI 1640 medium was supplemented with 2 mM glutamine (GE Healthcare/PAA, Little Chalfont, United Kingdom).

### Nuclease-probe

The nuclease-activated P2&3 TT-probe was developed in the McNamara lab of the Department of Internal Medicine, Roy J. and Lucille A. Carver College of Medicine, University of Iowa (USA) as previously described^28^. The probe was synthesized and HPLC purified by Integrated DNA Technologies (IDT) of Coralville, IA. The probe consists of the following: 5′-FAM-mU T T mU mU mU mU mU mU mU mU –ZEN-RQ-3′, where FAM is fluorescein amidite, mU is 2′-O-methyl modified U, T is unmodified deoxythymidine, ZEN is the ZEN quencher and RQ is the Iowa Black RQ quencher. The lyophilized probe was dissolved in 10 mM Tris-HCl pH 8.0, 1 mM EDTA to a final concentration of 500 µM and stored at −20 °C. For nuclease assays, 9 µL of 10 mM Tris-HCl pH 9.0, 10 mM CaCl_2_ was added to 1 µL 500 µM probe to yield the probe working stock. For reactions, 1 µL probe working stock was added to 9 µL substrate. Growth medium with added nuclease was used as negative control. For positive controls 1 µL purified nuclease (10 U/mL) was added. The reactions were incubated for 1 hour at 37 °C. Then 290 µL of 10 mM Tris-HCl pH 9.0, 10 mM EDTA was added to stop the activity of micrococcal nuclease by binding the free Ca^2+^ ions which are needed for nuclease activity. 95 µL of each stopped reaction was transferred to each of 3 wells of a flat bottomed 96-well plate and FITC fluorescence was measured.

### Reagents

TSB was prepared by dissolving 40 g Oxoid tryptone soya broth (CM0129) in 1 L purified water. LB was prepared by dissolving 25 g of Difuco LB Broth, Miller (Luria-Bertani) in 1 L of purified water. Recombinant micrococcal nuclease was purified from *Lactococcus lactis* as previously described^36^. Blood was donated by a healthy volunteer and stored in a BD Vacutainer® Blood Collection Sodium Citrate Tube, 0.105 M / 3.2%. Plasma was obtained by centrifuging the blood for 1 minute at 14000 rpm and extracting the plasma.

### Fluorescence measurements

All fluorescence levels were measured using a Biotek synergy 2.0 plate reader unless stated otherwise. Fluorescence was measured with filters optimal for FITC. Optics position was set at ‘bottom’ and sensitivity at 35.

### Nuclease activity and statistical analyses

Except where stated otherwise, the calculation of nuclease activity was carried out as follows. Mean fluorescence levels of negative control were subtracted prior to normalizations to correct for background interference. All data was normalized based on the mean positive control value. Normalization was based on the mean fluorescence of the positive control. Except where stated otherwise all incubations were executed in triplicate. All p-values were calculated using a Student’s t-test in IBM SPSS statistics 22.

### Secreted and cell wall-bound nuclease assay

To determine the effect of secreted and cell wall-bound nuclease on the nuclease probe activation, three varieties of *S*. *aureus* Newman were used, namely the wild-type strain, a Δ*nuc* mutant and a Δ*nuc* Δ*nuc*2 double mutant. The expression of the *nuc* gene leads to secretion of active nuclease. Expression of the *nuc*2 gene leads to expression of a cell wall-bound nuclease^37^. Probe incubated in TSB was used as negative control. Probe incubated in TSB plus purified recombinant nuclease were used as positive controls.

### Blood interference assay

To determine the effect of whole blood and plasma on the probe, the probe was suspended in either plasma or whole blood. From each suspension two samples were withdrawn and one sample was incubated with purified nuclease and one sample without. Probe incubated in PBS was used as a negative control and used to correct for background interference. Probe incubated in PBS and purified nuclease was used as positive control. After terminating the reaction, nuclease activity in the suspension was measured in triplicate.

### Sensitivity and specificity assays

To determine the sensitivity and specificity of the probe, all strains were grown on BA plates and 1 colony was incubated with the nuclease probe per protocol and subsequently imaged. Incubations were performed in triplicate. Negative controls were included in the analysis by incubating the probe in TSB or LB, while for positive controls purified nuclease was added to the TSB or LB. Correction for background was done using the negative controls in TSB or LB, depending on which growth medium was used to culture the bacteria. Normalization was performed based on the positive control in TSB or LB, depending on which growth medium was used to culture the bacteria.

For the growth curves, strains were cultured overnight in TSB, then washed 3 times with PBS. Optical Density (OD) was measured at 600 nm and all strains were resuspended at 0,0001 OD600 in TSB or RPMI 1640. After 0, 3, 6, 12, and 24 h of incubation, CFU were determined through serial dilution and plating in triplicate. Samples were collected and stored at −80 °C for subsequent nuclease activity measurements. This experiment was done three times.

### Ethical approval

The Independent Ethics Committee of the Foundation ‘Evaluation of Ethics in Biomedical Research’ (Assen, the Netherlands), approved the protocol for blood donations from healthy volunteers. This protocol is registered by QPS Groningen (code 04132-CS011). All volunteers provided their written informed consent. The study was performed with adherence to the guidelines of the Declaration of Helsinki and local regulations, and all samples were anonymized.

## Electronic supplementary material

Supplementary figure 1
