# Supplementary material for: Ex Vivo Tracer Efficacy in Optical Imaging of Staphylococcus Aureus Nuclease Activity
Source: Sci Rep. 2018 Jan 22;8:1305. doi: 10.1038/s41598-018-19289-y (PMC5778018; doi:10.1038/s41598-018-19289-y)
Supplement: Supplementary file 1 — Supplementary figure 1 [file 41598_2018_19289_MOESM1_ESM.doc]

**Supplementary Material**

**Figure 1**

***EX VIVO* TRACER EFFICACY IN OPTICAL IMAGING OF *STAPHYLOCOCCUS AUREUS* NUCLEASE ACTIVITY**

Colin W.K. Rosman^1,2,3^, Francisco Romero Pastrana^1^, Girbe Buist^1^, Marjolein Heuker^1^, Marleen van Oosten^1^, James O. McNamara^4^, Gooitzen M. van Dam^2#^, Jan Maarten van Dijl^1^

^1^Department of Medical Microbiology, University of Groningen, University Medical Center Groningen, Groningen, The Netherlands

^2^Department of Surgery, University of Groningen, University Medical Center Groningen, Groningen, The Netherlands

^3^Department of Biomedical Engineering, University of Groningen, University Medical Center Groningen, Groningen, The Netherlands

^4^Department of Internal Medicine, Roy J. and Lucille A. Carver College of Medicine, University of Iowa, Iowa City, Iowa, USA

**^#^Corresponding author:** Gooitzen M. Van Dam, University of Groningen, University Medical Center Groningen**,** Department of Surgery, Hanzeplein 1, PO Box 30001, 9700 RB Groningen, The Netherlands. Telephone: +31 (0)50 361 2301, E-mail: g.m.van.dam@umcg.nl

**Supplementary Figure 1**


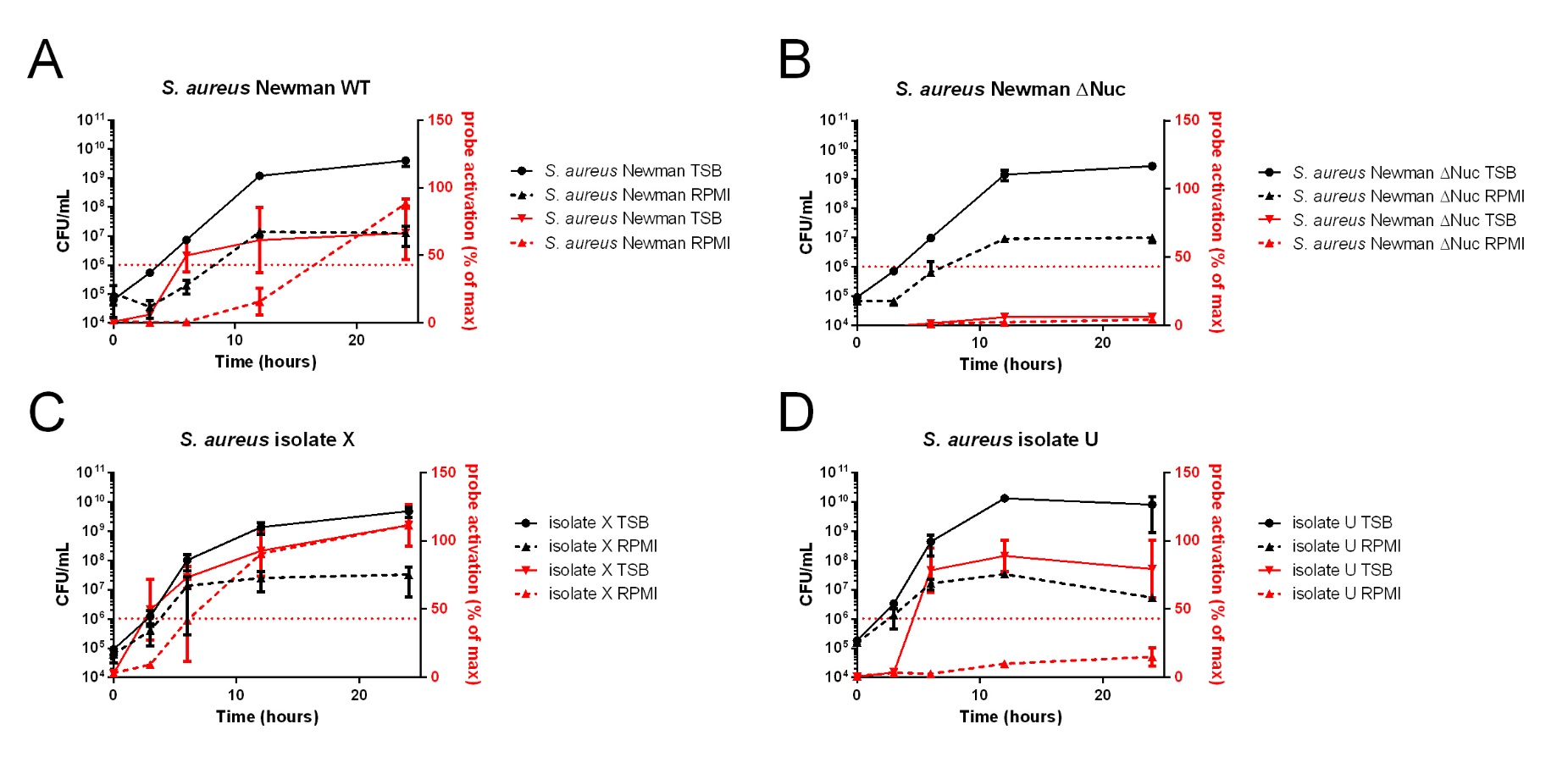


Activation of the poly T-probe by *S. aureus* grown to different stages in TSB and RPMI. The poly T probe consists of the same fluorophore and quencher as the P2&3 TT probe, but the connecting oligonucleotide consists of 11 thymine bases. This results in a higher sensitivity for nuclease activity, but lowers the specificity to micrococcal nuclease as this probe can be degraded by many different endonucleases^28^. The experiment was conducted using the same samples that were used for measurements with the P2&3 TT probe in Figure 4. Left Y-axis and lines in black indicate CFU per mL, right Y-axis and lines in red indicate probe activation as a percentage of the maximum activation. All experiments were done in triplicate, and the plots present the average numbers of the measurements. Capped lines indicate standard deviation. Strains included were: **A**, *S. aureus* Newman wild-type; **B**, nuclease deficient *S. aureus* mutant (Δ*nuc*); **C**, *S. aureus* clinical isolate X^29^; and **D**, *S. aureus* clinical isolate U^29^**.**
